# Synthesis of Zinc Oxide Nanoparticles From Cymodocea Serrulata Leaf Extract and Their Biological Activities

**DOI:** 10.7759/cureus.55521

**Published:** 2024-03-04

**Authors:** Vantipalli Raga Sai Harshitha, Ilangovar I.G.K, Vasugi Suresh, Sivaperumal Pitchiah

**Affiliations:** 1 Department of Physiology, Saveetha Dental College and Hospitals, Saveetha Institute of Medical and Technical Science, Saveetha University, Chennai, IND; 2 Department of Prosthodontics, Saveetha Dental College and Hospitals, Saveetha Institute of Medical and Technical Science, Saveetha University, Chennai, IND

**Keywords:** spectroscopy, antibacterial, zinc oxide nanoparticles(zno), leaf extract, cymodocea serrulata

## Abstract

Introduction

The utilization of *Cymodocea serrulata* for the eco-friendly synthesis of zinc oxide nanoparticles, which contain distinguishable nanostructures, presents a cost-effective and environmentally sustainable alternative for producing zinc nanoparticles. The production process of zinc nanoparticles are rich in phytochemicals, which can serve as stabilizing and reducing agents. Zinc nanoparticles can easily pass through bacterial cell walls and reach all cellular components. *C. serrulata*, is a small submerged angiosperm commonly found in submerged and tidal coastal environments.

Aim

Analysis of the biological activities of zinc oxide nanoparticles made from *C. serrulata* leaf extract.

Materials and Methods

Dry leaves of *C. serrulata* were ground into a powder, which was then placed into a conical flask and filled with water. Subsequently, the color of the mixture turned black. Next, a 20 mm piece of ZnO was dissolved in a 60 ml sample of distilled water to prepare the metal solution. Following this, a wavelength scan ranging from 200 to 700 nm was conducted using ultraviolet (UV) spectroscopy. After shaking the solution for an hour, a final reading was taken across the UV spectrum. The synthetic sample should also be centrifuged to remove any pellets and subsequently dried in a hot air oven.

Result

Using nanoscale profiling, the average particle size was measured and found to be less than 100 nm, specifically UV spectrum analysis revealed a notable absorbance value of 47.0 nm, at different angles within the peak height. The wavelength range of the zinc nanoparticles was observed to be between 250 and 350 nm.

Conclusion

The antibacterial properties of ZnO NPs have been demonstrated through in vitro investigations, indicating their potential application in in vivo studies.

## Introduction

Utilizing *Cymodocea serrulata *(*C. serrulata*), zinc oxide nanoparticles (ZnO NPs) are sustainably biosynthesized. Skin cancer, one of the most common cancers in humans, is accelerated by sunburns, which underscores the importance of this research. Nanoparticles, due to their distinct physicochemical characterization, hold greater significance than parent materials [1]. Identifiable nanostructures are present within them. Biosynthesis, a technique for creating nanoparticles with medicinal significance, employs bacteria and plants, offering a less expensive and environmentally sustainable alternative to physical and chemical procedures that utilize hazardous compounds, resulting in potentially harmful nanoparticles for medical applications. The abundant phytochemicals utilized in the production of zinc nanoparticles can serve as both stabilizing and reducing agents [2]. Due to their cost-effectiveness and versatile properties, ZnO NPs find applications in the medical industry, pharmaceutical products, drug carriers, and various other fields. Chronic conditions such as diabetes, cancer, and inflammation significantly contribute to the generation of free radicals, leading to oxidative damage. ZnO NPs, among the most important metal oxide nanoparticles, are widely utilized across disciplines owing to their distinctive physical and chemical attributes [3].

Moreover, ZnO NPs possess outstanding antibacterial, antimicrobial, and UV-blocking properties. Consequently, finished fabrics in the textile industry incorporating ZnO NPs exhibit desirable characteristics such as resistance to UV and visible light, antibacterial properties, and deodorizing effects. The antibacterial capabilities of ZnO NPs have garnered significant interest among scientists worldwide, particularly with the advent of nanotechnology enabling the production of particles at the nanoscale. Many bacteria range in size from hundreds of nanometers to tens of micrometers. ZnO NPs offer attractive antibacterial properties owing to their increased specific surface area and reduced particle size, enhancing particle surface reactivity [4].

The indiscriminate action of inorganic antibacterial drugs has led to a shift towards utilizing ZnO NPs to combat microbial resistance. The small particle size and large surface area of ZnO NPs can enhance surface reactivity, thereby increasing antibacterial activity. However, variations in the surface properties of nanomaterials may affect their interactions with cells, potentially compromising the intended antibacterial effect of ZnO NPs. Surface modifiers coating ZnO NPs may therefore play a crucial role in modulating antibacterial activity. In biosensing applications, ZnO NPs exhibit high catalytic efficiency, strong adsorption capability, a high isoelectric point, biocompatibility, and rapid electron transfer kinetics. Additionally, they find utility in various domains such as optical, piezoelectric, magnetic, and gas sensing [5]. *C*. s*errulata*, a marine submerged angiosperm, is commonly found in tidal and submerged coastal areas. Its meadows play a vital role in processing various ingredients, fostering marine biodiversity, regulating water, and providing benefits to humans. Research has also been conducted on the utilization of zinc oxide nanoparticles mediated by *C. serrulata* and their antioxidant, antibacterial, and cytotoxic properties. Nanoparticles, characterized by a high volume-to-surface area ratio, hold significant potential. The application of zinc oxide and other metal oxide nanoparticles in biomedical and cancer treatments is increasingly crucial due to their unique physical and chemical attributes [6]. Nanoparticles possess several distinct properties that make them promising agents for combating cancer. Studies on zinc oxide nanoparticles have revealed their potential for examining cancer cell apoptosis in detail, likely mediated by reactive oxygen species through the p53 pathway. This study aims to synthesize zinc nanoparticles using *C. serrulata* leaf extract and examine their biological properties [7].

## Materials and methods

Chemicals

The ZnO and Zn(NO_3_)_2_ chemical components were sourced from ground materials (from Sigma-Aldrich, USA, and Hi-Media Laboratory, India) known for their high analytical purity [8].

Extract preparation

**Figure 1:**
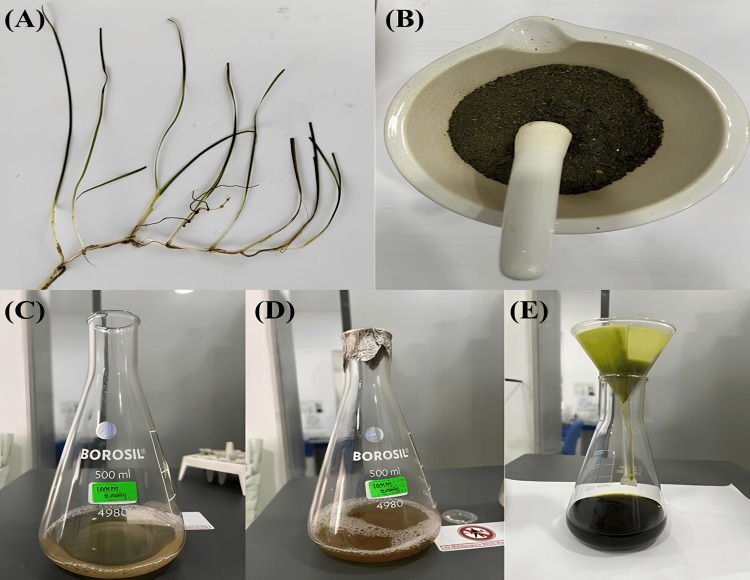
(A-E) Synthesis and preparation of ZnO NPs from C. serrulate ZnO NPs: zinc oxide nanoparticles; *C. serrulata*: *Cymodocea serrulate*

The dried leaves of *C. serrulata* (Figure 1A) were ground into a fine powder (Figure 1B). This powder was then placed into a conical flask and immersed in water (illustrated in Figures 1C-1D). Next, a 20 mm piece of Zn (NO_3_)_2_ was dissolved in a 60 ml sample of distilled water to create the metal solution. The resulting mixture was filtered, resulting in a noticeable color change to black (as seen in Figure 1E). Subsequently, ultraviolet (UV) spectroscopy was employed to conduct a wavelength scan ranging from 200 to 700 nm. After vigorous shaking of the solution for an hour, a final UV spectrum reading was taken. Furthermore, the synthesized sample underwent centrifugation to separate the pellet, which was then dried in a hot air oven [9].

Synthesis of ZnO NPs

An aqueous solution of Zn (NO_3_)_2_ (10 mM) was prepared using double-distilled water. Subsequently, 100 mL of the Zn (NO_3_)_2_ solution was transferred to a conical flask, and 5 to 10 mL of previously prepared aqueous extract was added dropwise on an orbital shaker while continuously stirring. The resulting biosynthesized solution was visually observed and further examined using a UV spectrophotometer with wavelengths ranging from 200 to 800 nm. Following this, the biosynthesized samples were centrifuged at 12,000 rpm. The resulting pellets were separated and then placed in a hot-air oven at 65°C for 24 hours [10].

Antibacterial activity of ZnO NPs

The antibacterial activity of ZnO NPs was evaluated using the disc diffusion method. Whatman filter paper discs (5 mm) were impregnated with various concentrations of NPs. Nutrient agar plates were inoculated with three common oral bacterial pathogens: *Klebsiella sp*., *Staphylococcus aureus,* and *Streptococcus mutans.* Wells were created on the agar plates using a sterile cork borer. To disperse the produced ZnO NPs uniformly, a measured quantity was dissolved in deionized water and then sonicated. ZnO NPs at concentrations of 75 and 100 μg/mL were added to the agar wells. The plates were then incubated at 37°C for 24 hours. The efficacy of ZnO NPs as an antibacterial agent against oral infections was assessed by measuring the diameters of their respective zones of inhibition. Means and standard deviations were calculated from three independent samples. Tetracycline (10 μg/disc) was used as a positive control [11]. An initial scan ranging from 200 to 700 nm was performed using a UV spectroscopy photometer. The final reading over the UV spectrum was taken after shaking the solution for an hour. The synthesized sample was centrifuged, the pellet was separated, and it was then dried in a hot air oven. Three different pathogens, including mutant *Staphylococcus*, MRSA, and *Klebsiella,* were selected due to their antibacterial properties. Consequently, three droplets of the liquid culture were placed on a plate containing these pathogens [11].

Green production of nanoparticles

A modified version of a previously published process was employed to synthesize zinc oxide. *C. serrulata *aqueous extract (20 ml) was continuously agitated with 0.005 M ZnCl_2_.7H_2_O for approximately 30 minutes. Subsequently, the solution was transferred to a 100-ml conical flask and heated to 70°C, followed by boiling with a magnetic stirrer until a brown precipitate formed, indicating the completion of the reaction. The resulting powder was centrifuged at 6000 rpm for 20 minutes, washed three times with ethanol and distilled water to isolate the pure product, and then dried in an oven at 80°C for six hours. Finally, the product was calcined at 450°C to obtain gray-coloured ZnO nanoparticles labeled as *C. serrulata*-ZnO [12].

Ultraviolet-visible spectroscopy

The optical properties of the generated ZnO NPs were determined from the absorption spectra obtained at various temperatures and concentrations. Characterization was performed using an ultraviolet-visible spectrometer with wavelengths ranging from 200 to 800 nm [13,14].

## Results

UV-visible spectroscopy of ZnO NPs

**Figure 2:**
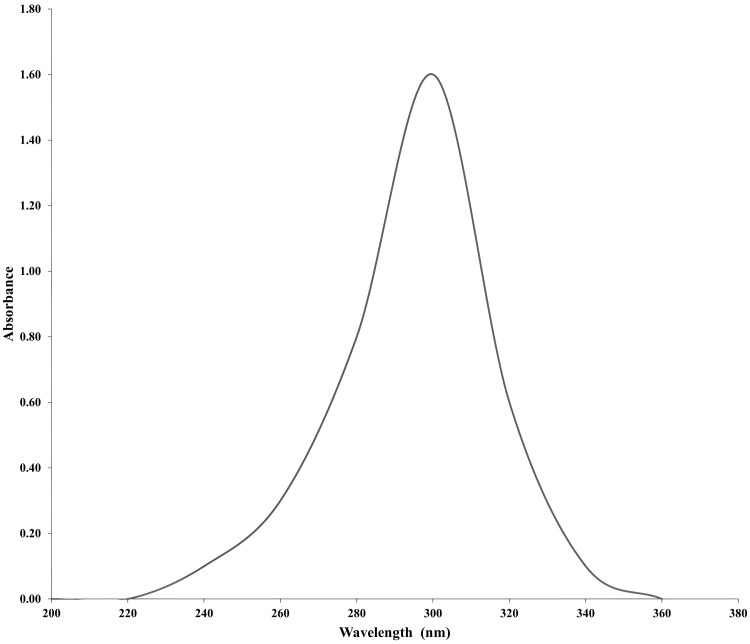
UV visible spectroscopy of ZnO NPs. UV: ultraviolet; ZnO NPs: zinc oxide nanoparticles

The UV spectrum graph in Figure 2 shows that the ZnO NPs synthesized from *C. serrulata* leaves exhibited a maximum absorbance of 1.6 at a wavelength of 300 nm. 

Antibacterial activity

**Figure 3:**
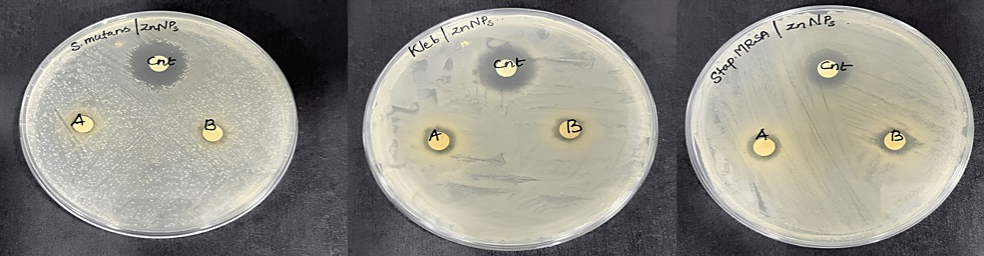
(A) The zone of inhibition of C. serrulata against S. mutans, (B) The zone of inhibition of C. serrulata against Klebsiella sp., and (C) The zone of inhibition of C. serrulate against S. aureus. * C. serrulate*: *Cymodocea serrulate*,* S. mutans*:* Staphylococcus mutans*,* S. aureus*:* Streptococcus aureus*

The antibacterial activity of green-synthesized ZnO NPs against three different oral pathogens, *S. mutans*, *Klebsiella sp*., and *S. aureus*, was assessed by measuring their inhibition zones around the discs from the back of the plate (see Figure 3). The NPs exhibited excellent antibacterial activity at two concentrations, with inhibition zones measuring 7.5 ± 0.5 mm, 9.5 ± 1.2 mm, and 10 ± 1.2 mm for *S. mutans*, *Klebsiella sp*., and *S. aureus*, respectively, at a ZnO NP concentration of 100 μg/mL. Furthermore, at a concentration of 75 μg/mL, the inhibition zones were 7 ± 0.5 mm, 8 ± 1 mm, and 9 ± 1 mm for the respective bacteria (refer to Table 1).

**Table 1:** Inhibition zone by ZnO NPs on three different oral pathogens: S. mutans, Klebsiella sp., S. aureus (MRSA). ZnO NPs: zinc oxide nanoparticle; *S. mutans*: *Streptococcus mutans*; *S. aureus*: *Staphylocoocus aureus;* MRSA: methicillin-resistant *Staphylococcus aureus*

Nanoparticle concentration (μg/mL)	Streptococcus mutans (mm)	Klebsiella sp. (mm)	Staphylococcus aureus (MRSA) (mm)
100	7.5 ± 0.5	9.5 ± 1.2	10 ± 1.2
75	7 ± 0.5	8 ± 1	9 ± 1

## Discussion

The maritime environment serves as a remarkable source of potent biological activity. Utilizing microbes and plants for nanoparticle synthesis presents a safe, environmentally responsible, biocompatible, and economically viable option. Plants are considered the preferred source for mass manufacturing stable nanoparticles [15]. Metal ions are reduced by phytochemicals found in plants, including polyphenols, polysaccharides, terpenoids, alkaloids, vitamins, and amino acids [16]. *C. serrulata* is medicinally utilized for various conditions, such as malaria treatment, and as a sedative for infants and pregnant women. Seagrass contains phytoconstituents capable of altering the size, shape, content, and physicochemical characteristics of nanoparticles. In our study, a zinc acetate solution effectively synthesized ZnO NPs from seagrass extract, resulting in a black precipitate settling at the bottom of the vessel upon the addition of distilled water [17]. The phytomolecules in seagrass serve to reduce and stabilize zinc nanoparticles. Confirmation of zinc nanoparticle synthesis was obtained through UV spectroscopy, with absorption peaks observed between 250 and 700 nm. Our zinc oxide nanoparticles exhibited an absorption peak around 350 nm. Similarly, comparable high UV absorption peaks were found in ZnO nanoflakes synthesized using *C. serrulata* leaf extract. The presence of zinc elements in the zinc oxide nanoparticles mediated by *C. serrulata* was further confirmed by X-ray diffraction data. Atomic force microscopy was employed to measure the zinc oxide nanoparticles, providing a three-dimensional height profile for accurate sample measurement [18].

A study reported a maximum absorbance peak at 370 nm for ZnO NPs synthesized using *Pelargonium odoratissimum* aqueous leaf extract (ALE) [19]. Observation of the inhibition zones of A. marina-synthesized ZnO NPs revealed the most significant antibacterial activity against *Klebsiella sp*. at 100 μg/mL and *S. aureus* at 75 μg/mL, with inhibition zones of 9.5 ± 1.2 mm and 9 ± 1 mm, respectively. Overall, *S. aureus* exhibited the highest inhibition at both 100 and 75 μg/mL, with inhibition zones of 9.5 ± 0.5 mm and 9 ± 1 mm, respectively. Previous studies reported that biosynthesized ZnO NPs from *Pseudomonas aeruginosa* exhibited high efficacy against S. aureus, with an inhibition zone of 12.33 ± 0.9 mm [20]. In contrast, A. marina-mediated ZnO NPs showed inhibition zones of 9.5 ± 0.5 mm and 9 ± 1 mm for two different concentrations of *S. aureus*. Another study found that biosynthesized silver NPs from the leaf extract of *A. marina* exhibited an inhibition zone of only 10.87 ± 1.33 mm against* S. aureus* [21]. Similarly, ZnO NPs synthesized from the same plant demonstrated inhibition zones for three pathogens: *S. aureus*, *S. mutans*, and *Klebsiella sp.* (9.5 ± 0.5 mm, 9 ± 1 mm), (7.5 ± 0.2 mm, 7 ± 0.25 mm), and (7.5 ± 0.2 mm, 7 ± 0.25 mm), respectively. Furthermore, a study showed that Ag/Fe2O3 NPs at 5 g/mL had a significant antibacterial effect on *S. aureus*, with an inhibition zone of 22.3 ± 0.57 mm [22]. Similarly, in another study, copper NPs synthesized from *Kigelia africana* fruit exhibited a striking inhibition zone of 8.0 ± 2.83 mm on *S. aureus* [23]. Using the Mueller-Hinton agar method, one study demonstrated that platinum NPs prepared using *Atriplex hamilus* leaves had an inhibition zone of 17 mm for *Klebsiella pneumonia *[24]. Finally, ZnO NPs synthesized using* P. odoratissimum* leaf extract exhibited a maximum inhibition zone of 28 ± 0.35 mm for *S. aureus* at a concentration of 10 μg/mL [25].

Limitations

This study was confined to only three oral pathogens, with a limited sample size. There is a pressing need to evaluate these biosynthesized ZnO NPs against a broader spectrum of microorganisms present in the oral mucosa. Furthermore, detailed descriptions of the characterization techniques employed to confirm the synthesis of ZnO NPs, such as Fourier transform infrared spectroscopy (FTIR), scanning electron microscopy (SEM), and X-ray diffraction (XRD), are crucial. Without comprehensive investigation, it is challenging to ascertain the quality, size, shape, and purity of NPs. Moreover, the absence of in vivo testing limits our understanding of how these NPs interact within a living organism. It would be beneficial to explore potential adverse effects, tissue reactions, and actual efficacy within the oral cavity.

## Conclusions

The present study focuses on the green synthesis of ZnO NPs using the aqueous extract of *C. serrulata*, followed by analysis of the nanoparticles. A recorded absorbance value of 1.6 was observed at a wavelength of 300 nm in the UV spectrum. The diameter of the inhibition zones was measured to assess the antibacterial activity against *S. mutans*, *Klebsiella sp*., and *S. aureus*. At a concentration of 100 μg/mL ZnO NPs, the inhibition zones for *S. mutans*, *Klebsiella sp*., and *S. aureus* were measured at 7.5 ± 0.5 mm, 9.5 ± 1.2 mm, and 10 ± 1.2 mm, respectively. Similarly, at a concentration of 75 μg/mL, the bacterial strains exhibited inhibitory zones of 7 ± 0.5 mm, 8 ± 1 mm, and 9 ± 1 mm, respectively. The antibacterial properties of ZnO NPs have been demonstrated through in vitro investigations, suggesting their potential application in in vivo studies. Given their notable efficacy, ZnO NPs are promising for utilization in the pharmaceutical industry, particularly for drug delivery.
